# Supplementary figures and images for: Quantifying the fitness effects of resistance alleles with and without anthelmintic selection pressure using Caenorhabditis elegans
Source: PLoS Pathog. 2024 May 20;20(5):e1012245. doi: 10.1371/journal.ppat.1012245 (PMC11142691; doi:10.1371/journal.ppat.1012245)

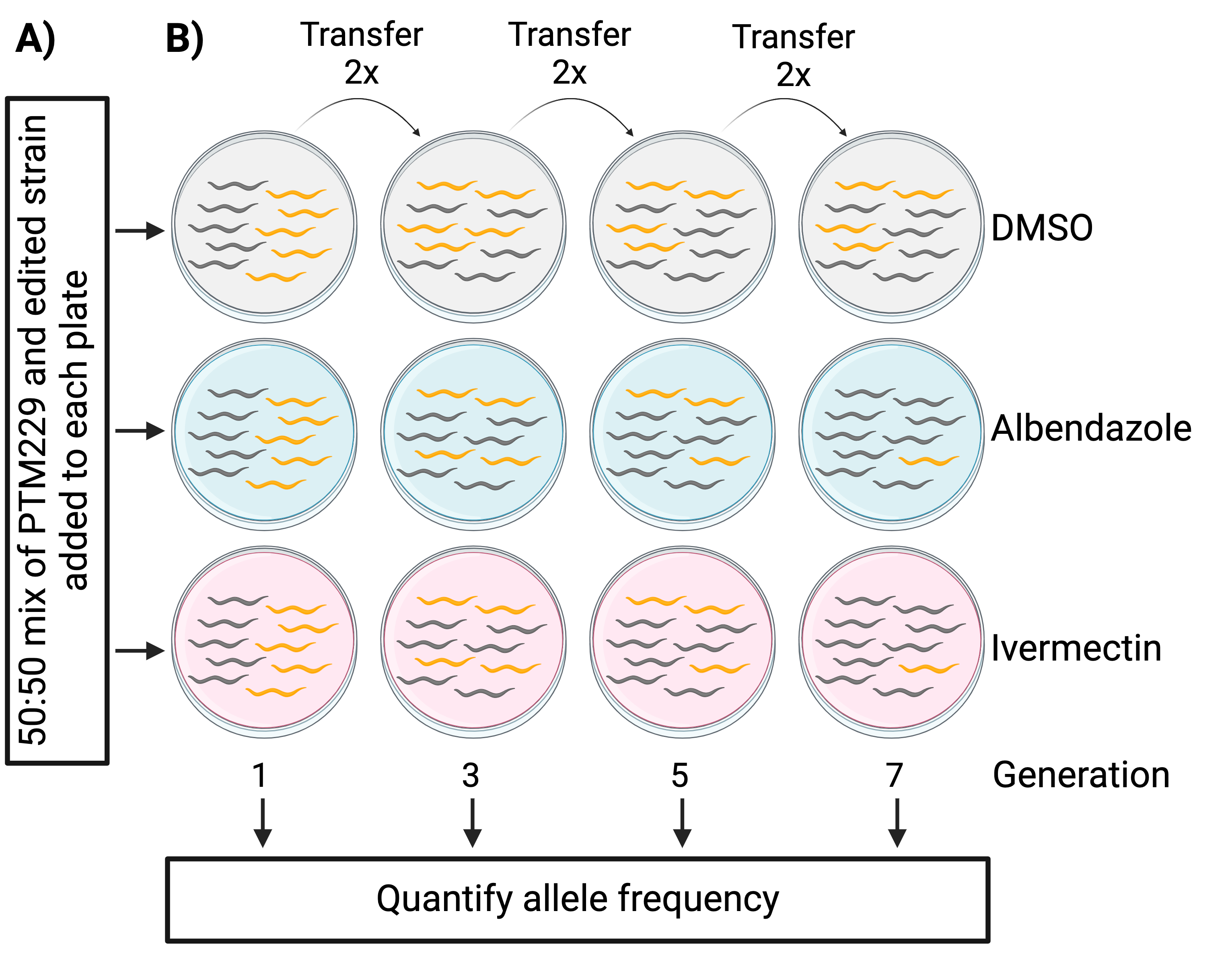

Supplement: S1 Fig — (A) Equal numbers of the control strain PTM229 were placed on each test plate along with an edited strain. (B) Strains were grown on 6 cm NGMA plates for approximately seven days. After seven days, a ~0.5 cm3 plate chunk of NGMA with animals was transferred to a new 6 cm NGMA plate. Animals were washed off of NGMA plates at each odd generation. After animal collection, DNA extractions, DNA cleanup, and quantification were performed. Allele frequencies were quantified using ddPCR. See Methods, Competitive fitness assays for details. Modified from a previous version [73]. Created with Biorender.com. (TIF) [file ppat.1012245.s009.tif]

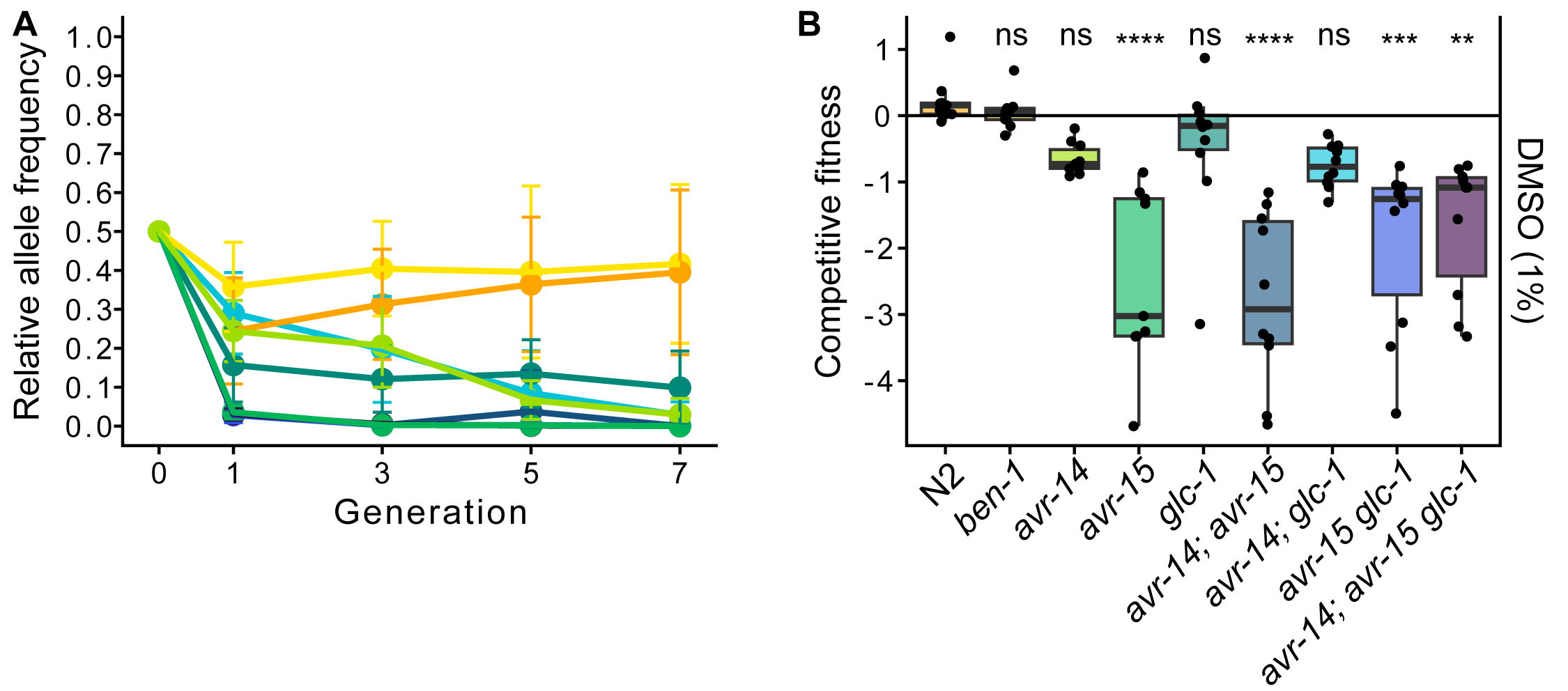

Supplement: S2 Fig — (A) A barcoded N2 wild-type strain, PTM229, was competed with strains that have deletions in either one, two, or three genes that encode for GluCl channels or in the beta-tubulin gene ben-1 in DMSO. Generation is shown on the x-axis, and the relative allele frequencies of the nine strains with genome-edited alleles and N2 are shown on the y-axis. (B) The log2-transformed competitive fitness of each allele is plotted in DMSO. The gene tested is shown on the x-axis, and the competitive fitness is shown on the y-axis. Each point represents a biological replicate of that competition experiment. Data are shown as Tukey box plots with the median as a solid horizontal line, and the top and bottom of the box representing the 75th and 25th quartiles, respectively. The top whisker is extended to the maximum point that is within the 1.5 interquartile range from the 75th quartile. The bottom whisker is extended to the minimum point that is within the 1.5 interquartile range from the 25th quartile. Significant differences between the wild-type N2 strain and all the other alleles are shown as asterisks above the data from each strain (p > 0.05 = ns, p < 0.001 = ***, p < 0.0001 = ****, Tukey HSD). (TIF) [file ppat.1012245.s010.tif]

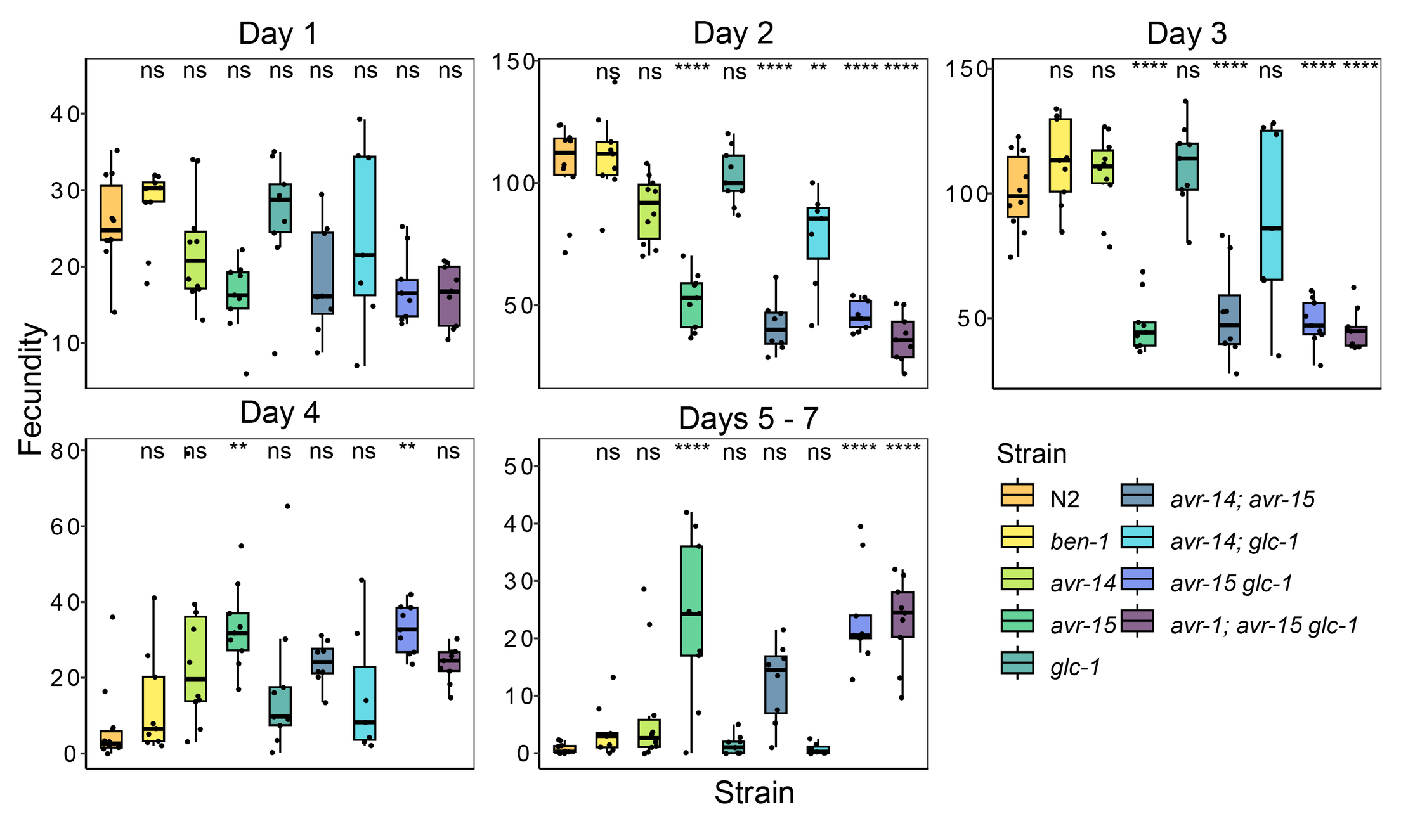

Supplement: S3 Fig — Boxplots for daily fecundity when exposed to DMSO on the y-axis, for each deletion strain on the x-axis. Each point represents the daily fecundity count for one biological replicate. Error bars show the standard deviation of lifetime fecundity among 7–10 biological replicates. Data are shown as Tukey box plots with the median as a solid horizontal line, and the top and bottom of the box represent the 75th and 25th quartiles, respectively. Significant differences between the wild-type strain, N2, and all other deletions are shown as asterisks above the data from each strain (p > 0.05 = ns, p < 0.05 = *, p < 0.01 = **, p < 0.001 = ***, p < 0.0001 = ****, Tukey HSD). (TIF) [file ppat.1012245.s011.tif]

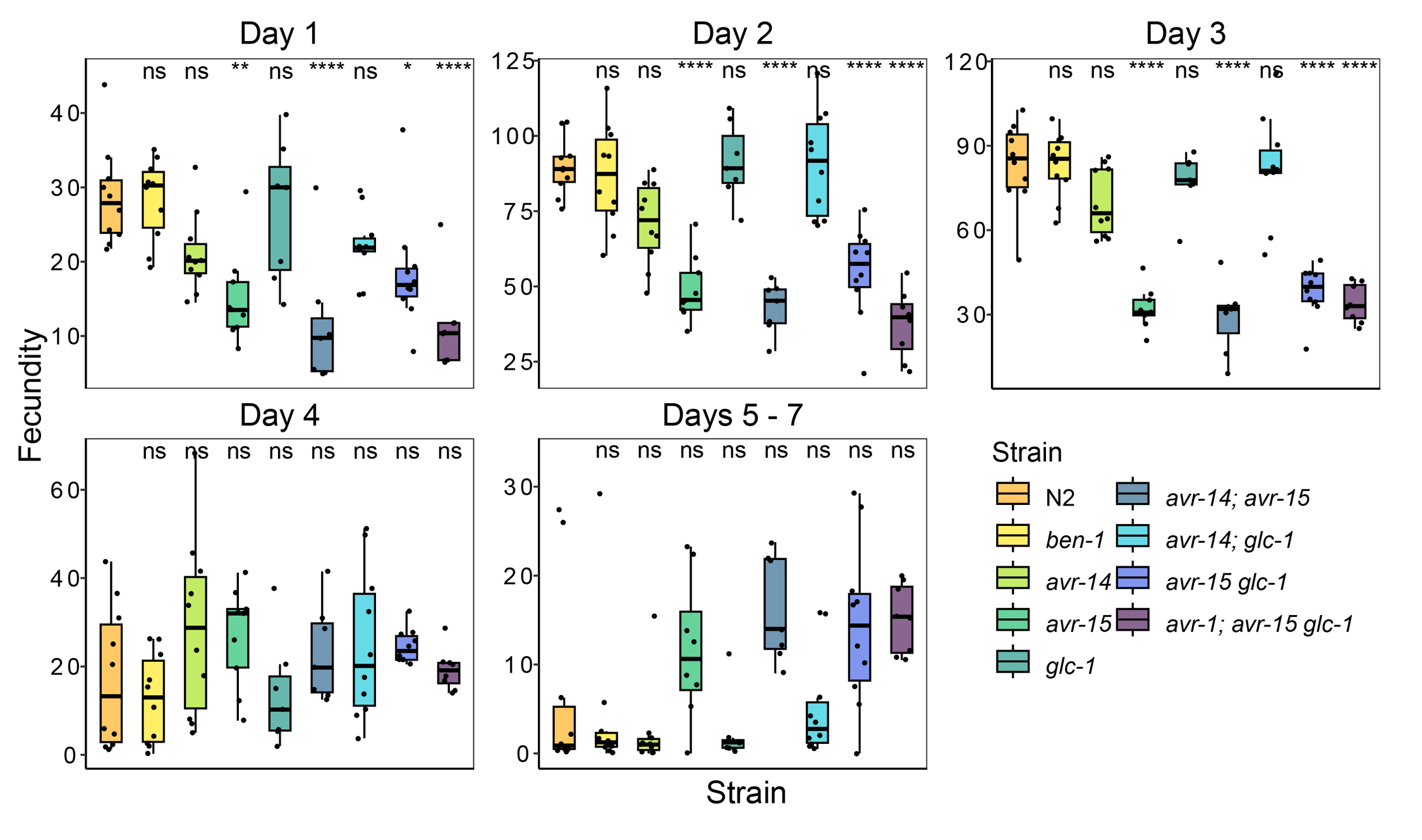

Supplement: S4 Fig — Boxplots for daily fecundity when exposed to albendazole on the y-axis, for each deletion strain on the x-axis. Each point represents the daily fecundity count for one biological replicate. Error bars show the standard deviation of lifetime fecundity among 7–10 biological replicates. Data are shown as Tukey box plots with the median as a solid horizontal line, and the top and bottom of the box represent the 75th and 25th quartiles, respectively. Significant differences between the wild-type strain, N2, and all other deletions are shown as asterisks above the data from each strain (p > 0.05 = ns, p < 0.05 = *, p < 0.01 = **, p < 0.001 = ***, p < 0.0001 = ****, Tukey HSD). (TIF) [file ppat.1012245.s012.tif]

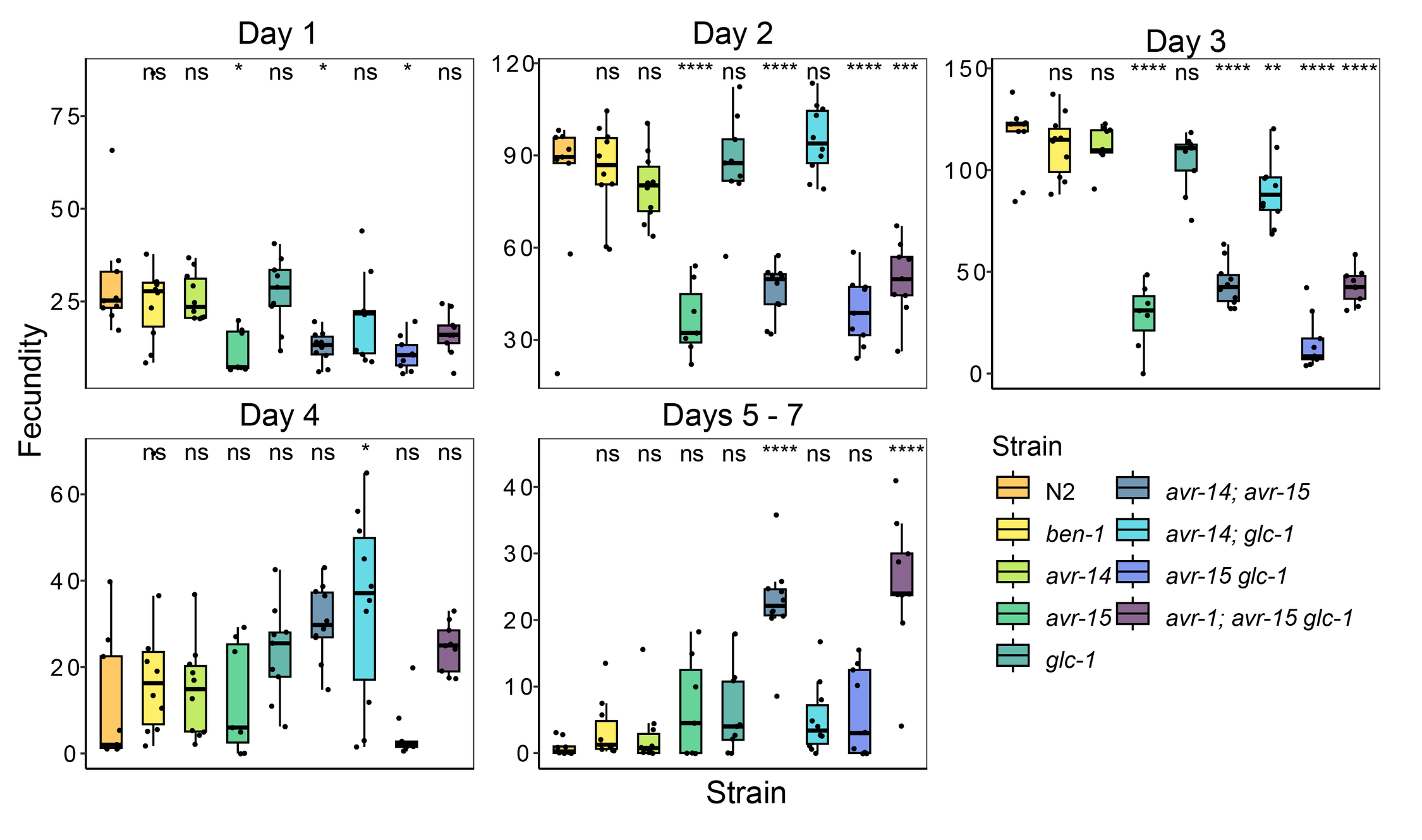

Supplement: S5 Fig — Boxplots for daily fecundity when exposed to ivermectin on the y-axis, for each deletion strain on the x-axis. Each point represents the daily fecundity count for one biological replicate. Error bars show the standard deviation of lifetime fecundity among 7–10 biological replicates. Data are shown as Tukey box plots with the median as a solid horizontal line, and the top and bottom of the box represent the 75th and 25th quartiles, respectively. Significant differences between the wild-type strain, N2, and all other deletions are shown as asterisks above the data from each strain (p > 0.05 = ns, p < 0.05 = *, p < 0.01 = **, p < 0.001 = ***, p < 0.0001 = ****, Tukey HSD). (TIF) [file ppat.1012245.s013.tif]

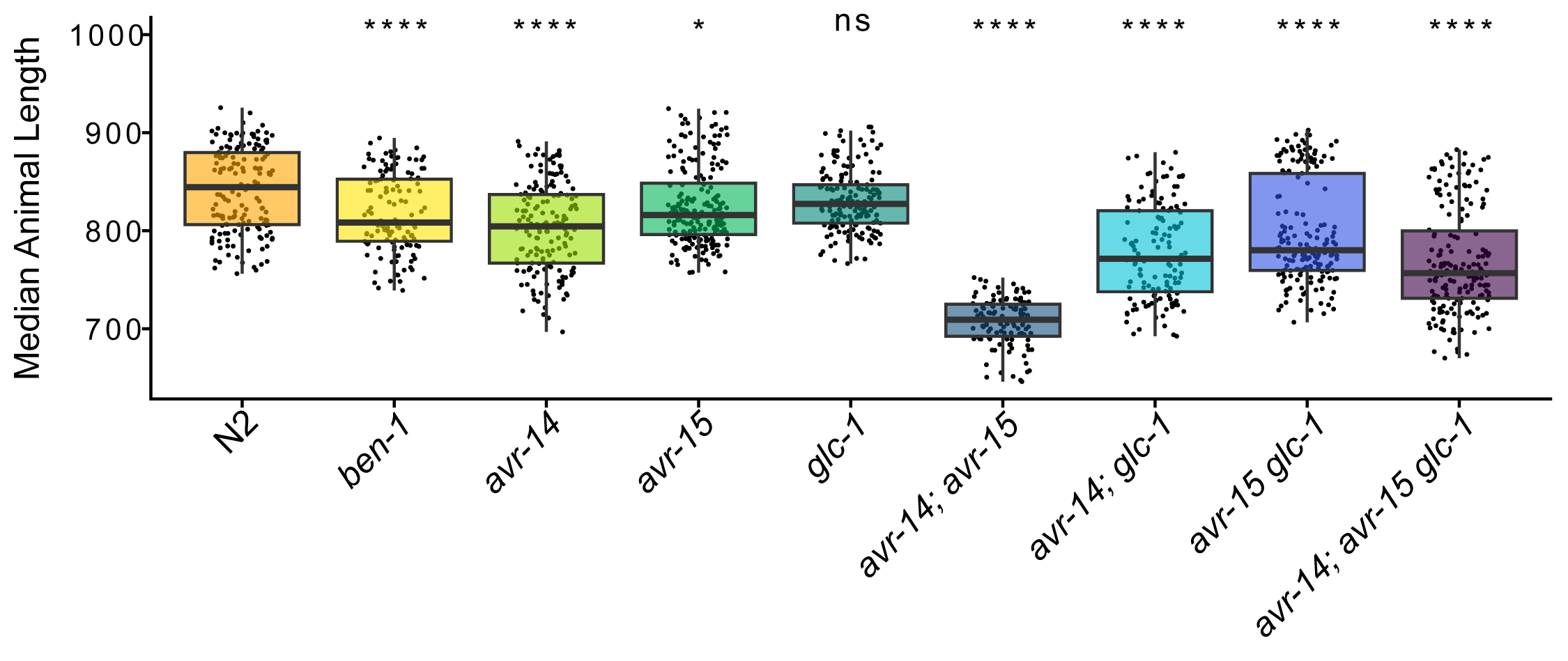

Supplement: S6 Fig — Median animal length values from populations of nematodes grown in DMSO are shown on the y-axis. Each point represents the median animal length from a well containing approximately 5–30 animals. Data are shown as Tukey box plots with the median as a solid horizontal line, the top and bottom of the box representing the 75th and 25th quartiles, respectively. The top whisker is extended to the maximum point that is within 1.5 interquartile range from the 75th quartile. The bottom whisker is extended to the minimum point that is within 1.5 interquartile range from the 25th quartile. Significant differences between the wild-type strain and all other strains are shown as asterisks above the data from each strain (p > 0.05 = ns, p < 0.05 = *, p < 0.001 = ***, p < 0.0001 = ****, Tukey HSD). (TIF) [file ppat.1012245.s014.tif]

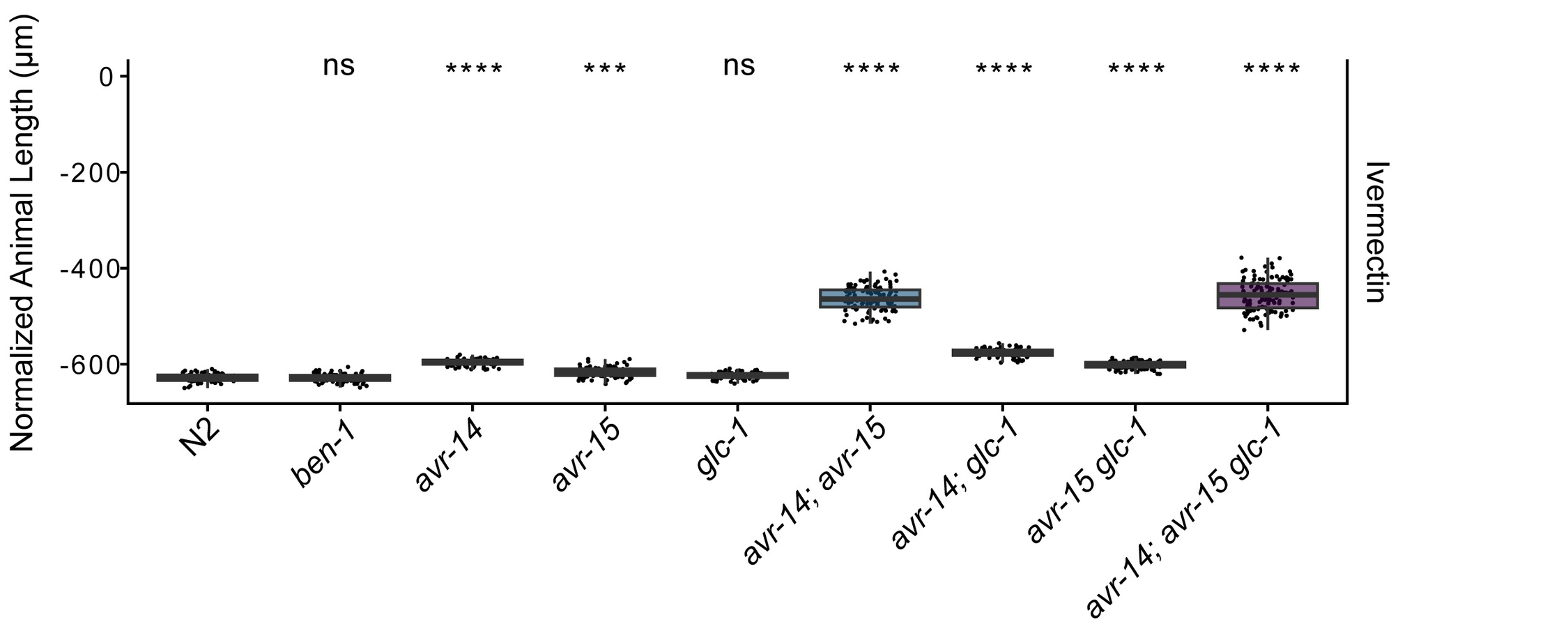

Supplement: S7 Fig — The regressed median animal length values for populations of nematodes growth in 500 nM ivermectin are shown on the y-axis. Each point represents the regressed median animal length value of a well containing approximately 5–30 animals. Data are shown as Tukey box plots with the median as a solid horizontal line, and the top and bottom of the box representing the 75th and 25th quartiles, respectively. The top whisker is extended to the maximum point that is within the 1.5 interquartile range from the 75th quartile. The bottom whisker is extended to the minimum point that is within the 1.5 interquartile range from the 25th quartile. Significant differences between the wild-type strain and all other deletions are shown as asterisks above the data from each strain (p > 0.05 = ns, p < 0.001 = ***, p < 0.0001 = ****, Tukey HSD). (TIF) [file ppat.1012245.s015.tif]

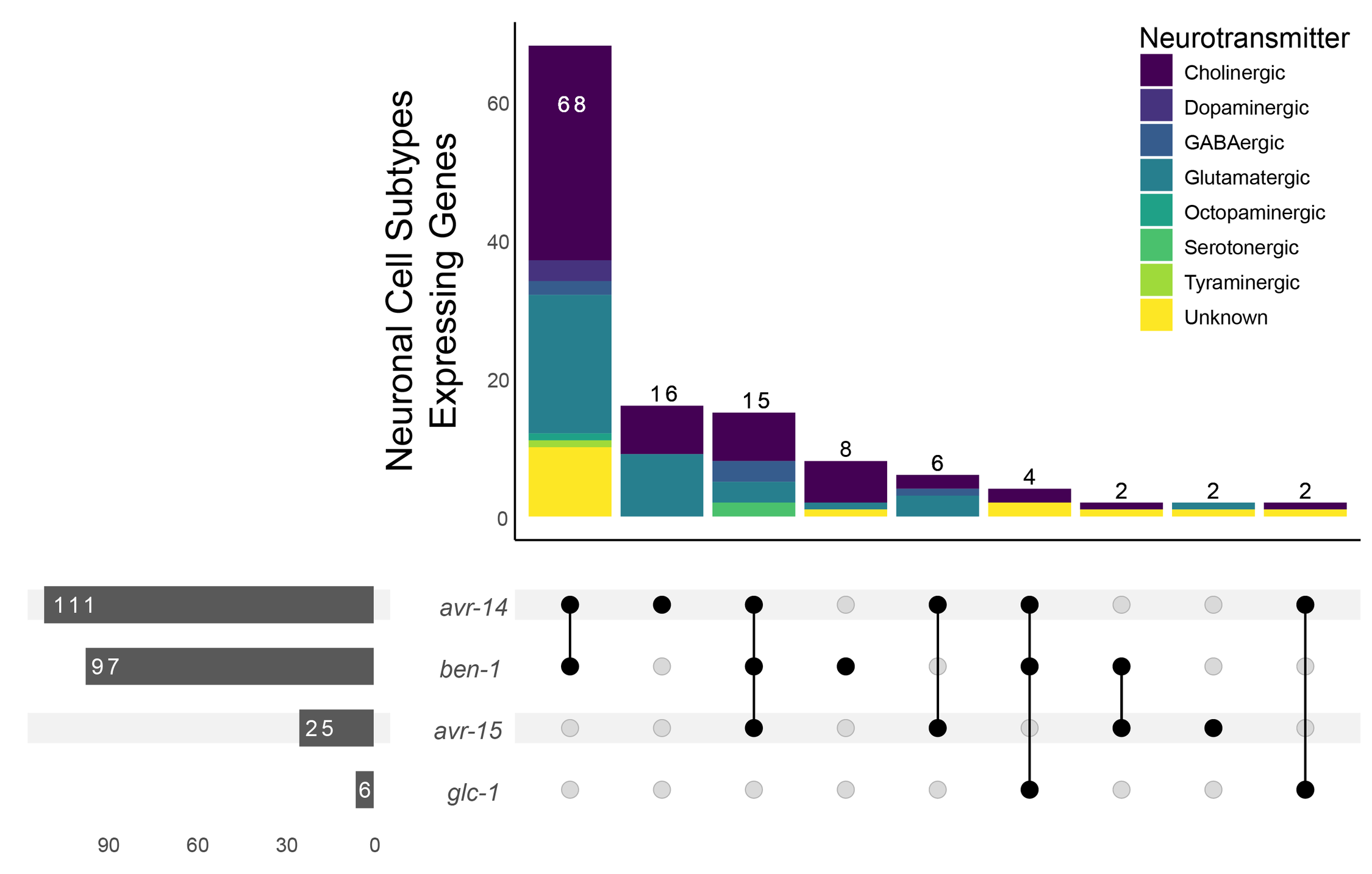

Supplement: S8 Fig — Upset plot of single-cell RNA-sequencing data obtained from CeNGEN. Horizontal bar plots sum the total number of neurons where the gene is expressed. Vertical bar plots sum overlap where genes are expressed in the neuronal cell subtypes. Black dots directly under vertical bar plots signify the gene(s) that overlap in the neuronal cell subtypes indicated in the vertical bar plot. (TIF) [file ppat.1012245.s016.tif]

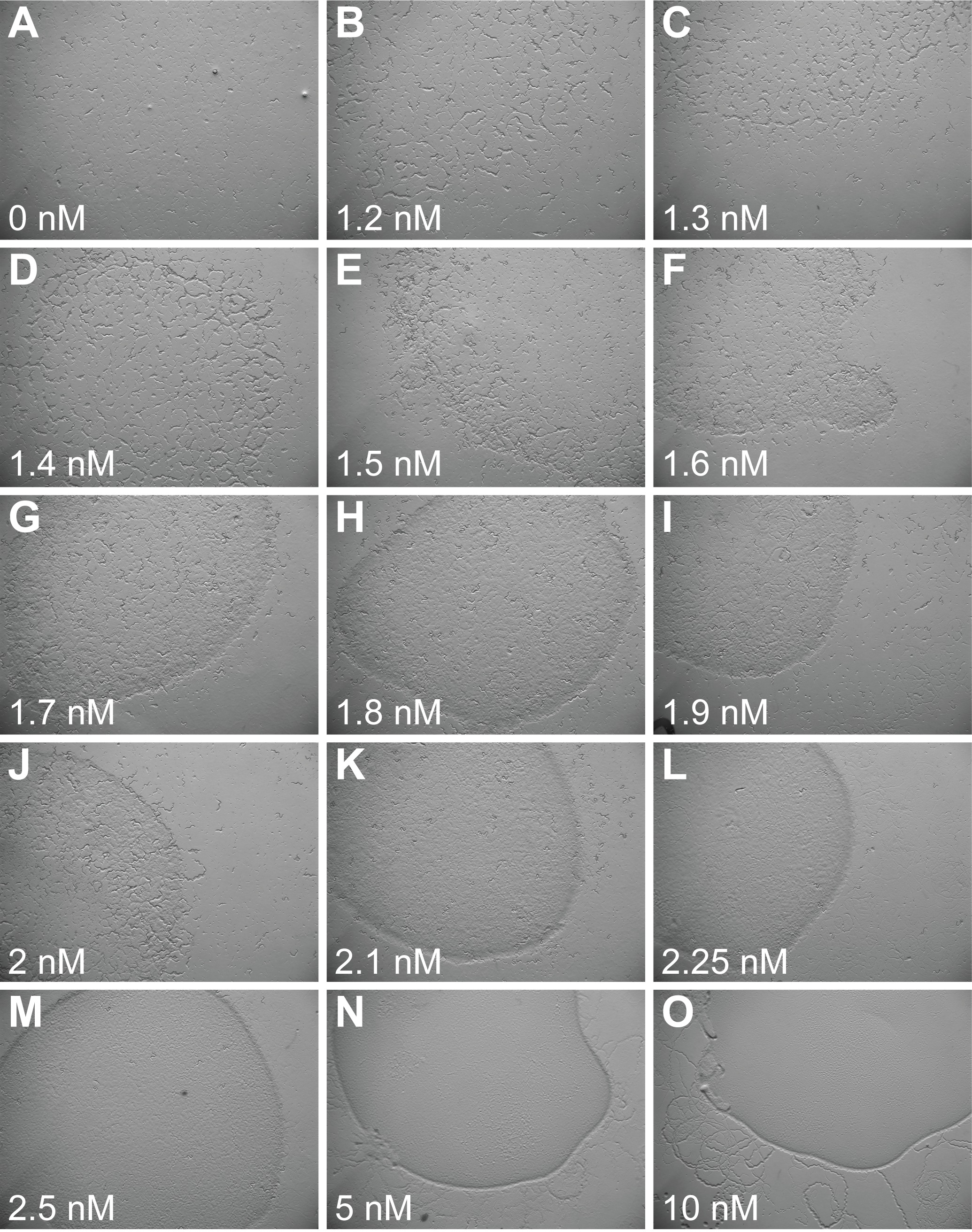

Supplement: S9 Fig — The single deletion strain avr-15 was exposed to ivermectin in a dose response manner. Animals were exposed to ivermectin at (A) 0 nM (1% DMSO), (B) 1.2 nM, (C) 1.3 nM, (D) 1.4 nM, (E) 1.5 nM, (F) 1.6 nM, (G) 1.7 nM, (H) 1.8 nM, (I) 1.9 nM, (K) 2.1 nM, (L) 2.25 nM, (M) 2.5 nM, (N) 5 nM, and (O) 10 nM. (TIF) [file ppat.1012245.s017.tif]

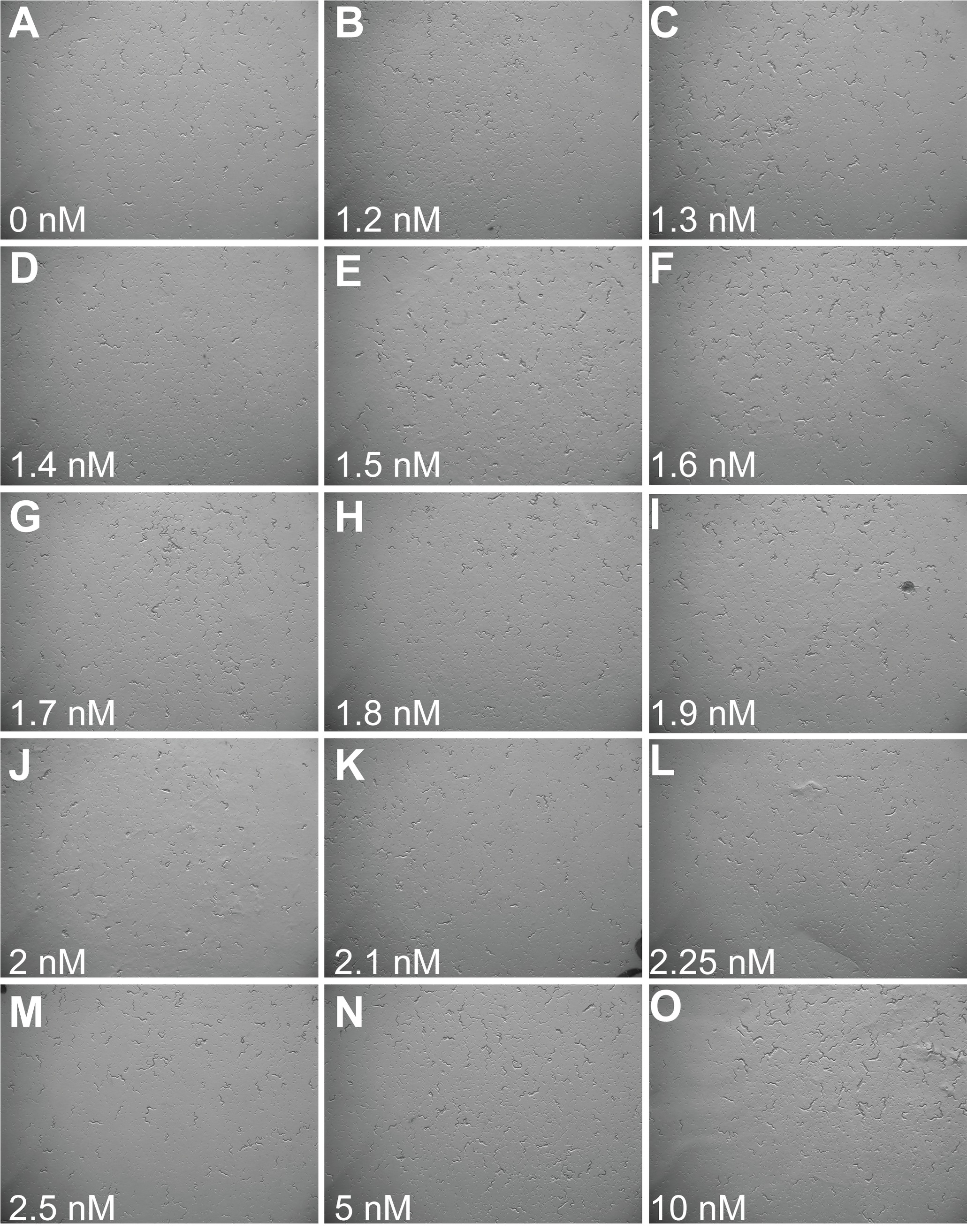

Supplement: S10 Fig — The triple mutant deletion strain (avr-14, avr-15, and glc-1) was exposed to ivermectin in a dose response manner. Animals were exposed to ivermectin at (A) 0 nM (1% DMSO), (B) 1.2 nM, (C) 1.3 nM, (D) 1.4 nM, (E) 1.5 nM, (F) 1.6 nM, (G) 1.7 nM, (H) 1.8 nM, (I) 1.9 nM, (K) 2.1 nM, (L) 2.25 nM, (M) 2.5 nM, (N) 5 nM, and (O) 10 nM. (TIF) [file ppat.1012245.s018.tif]
