# Supplementary material for: Quantifying the fitness effects of resistance alleles with and without anthelmintic selection pressure using Caenorhabditis elegans
Source: PLoS Pathog. 2024 May 20;20(5):e1012245. doi: 10.1371/journal.ppat.1012245 (PMC11142691; doi:10.1371/journal.ppat.1012245)
Supplement: Attachment — Submitted filename: BZML_Response_to_PLOS_Pathogens_review.pdf [file ppat.1012245.s019.pdf]

Dear Dr. Tim,

Thank you for handling our manuscript, “Quantifying the fitness effects of resistance alleles with and without anthelmintic selection pressure using *Caenorhabditis elegans*.” We have addressed all of the editor’s and reviewers' critiques, and the manuscript is vastly improved. Please find their comments in bold below with our responses in normal font. Changes to the manuscript are included as quotes below our responses.

Editor’s comments to the author:

1) Three qualified reviewers have offered markedly different recommendations for disposition of this manuscript. All agree that it is well-written and that it contains important information, although one reviewer questioned the relevance of the data for parasitic nematodes. After carefully reading the manuscript and the reviews, I believe the proper course of action is to request a revision in which each of the comments raised in the review process are thoroughly addressed or refuted. The most significant and common concern is that null mutations in AVR receptors have not been encountered in field-derived populations of parasitic nematodes resistant to macrocyclic lactones, which requires a thoughtful response from the authors. I believe that most of the other concerns can be more readily resolved.

> Thank you for the comments and the opportunity to resubmit our manuscript. As suggested, we have addressed each of the reviewers' concerns. We hope that our edits adequately address each comment and collectively address each reviewer’s concerns about null mutations in AVR receptors. As pointed out below, we did offer several explanations for why AVR mutations have not been encountered in ML-resistant field populations of parasitic nematodes (*i.e.*, variation in GluCl gene family number across species, interactions between genes outside the GluCl family, poor nematode genome quality, and the locations of GluCl tissue expression). We tried to amplify these reasons.

Part I - Summary

2) Reviewer #1: The manuscript by Shaver and colleagues describes the use of a C. elegans model to explore the phenotypes conferred by loss of function mutations in known anthelmintic target genes, with and without drug exposure.

I found the approach and resulting data very interesting – the competitive fitness assays and comparisons between wild isolates were especially well done. The finding of significant fitness costs associated with loss of function mutations in the GluCl subunits is a nice observation that has long been speculated to be the case, and fits with recent findings that these targets are not implicated in resistance in parasitic nematodes. However, I felt the introduction and discussion could be improved as the relevance and suggested implications for resistance in parasitic nematodes were not always well reasoned. I have only minor edits for the results section and methods, but I suggest the introduction and discussion should be re-drafted for clarity and accuracy.

> Thank you for the critique. We believe that the *Discussion* directly speaks to the relevance and implications for parasitic nematodes. Specifically, in the *Discussion*, we have a paragraph that speaks to each of the following topics: GluCl gene family number, interactions between genes outside the GluCl family, poor nematode genome quality, and locations of GluCl tissue expression.

We have made edits to the *Introduction* to address the reviewer's comments and ensure that readers understand why GluCls mutations have not been associated with ivermectin resistance in parasitic nematodes:

"However, the relationship between GluCls and the MoR of ivermectin is poorly understood. To date, mutations in GluCl subunits have been infrequently detected and associated with ivermectin-resistant parasitic nematodes. Our ability to find GluCl mutations in nature can be complicated by the variation in GluCl gene family number across species, interactions between genes outside the GluCl family, poor nematode genome quality leading to false negatives, and the locations of GluCl tissue expression (see *Discussion*)."

We hope these changes address the reviewer's concerns.

3) Reviewer #2: The manuscript aims at understanding the mechanisms and consequences of resistance to albendazole and ivermectin drugs, especially when administered together. While Albendazole resistance is mainly linked to variations in the beta-tubulin gene, ivermectin targets GluCl genes, but it is unknown whether GluCl genes are involved in ivermectin resistance in nature. Therefore, the study, conducted with Caenorhabditis elegans, investigated the fitness costs associated with the loss of drug target genes. The results revealed that albendazole resistance required the deletion of the beta-tubulin gene ben-1, while ivermectin resistance necessitated the loss of two (avr-14 and avr-15) or three (avr-14, avr-15, and glc-1) GluCl genes. Fecundity assays showed no fitness benefit in albendazole with the loss of ben-1, and no GluCl mutants were resistant to ivermectin. The study also explored evidence of multi-drug resistance across different traits, finding no evidence that loss of ben-1 or any GluCl subunit conferred cross-resistance.

The manuscript is well written and the materials and methods is very detailed. Moreover, the high-throughput assay (HTA), rigorously conducted, is an original way of assessing nematode fitness. While the conclusions about benzimidazole resistance are very clear, the relevance of the results on ivermectin resistance is less so. Indeed, while the authors findings provide insights into why GluCls have not been clearly associated with resistance in parasites, it does not answer how resistance alleles could spread in parasitic nematodes populations since mutations of GluCl are not found in selected ivermectin resistant strains, although the authors have set themselves the goal of answering this question. Instead, the authors could have used established resistant strains like the IVR10 (James et al. International Journal for Parasitology, 2009) or strains deleted for genes required for amphids formation that are naturally resistant to MLs (such as the dyf-7 deleted strain).

Thank you for the critique.

- Our goal in this manuscript was not to assess how all genes implicated in ivermectin resistance respond in our assays but to instead quantitatively assess how GluCls interact with and without drug pressure. We agree that additional strains that target additional genes outside of GluCl subunits would be informative to understand ivermectin resistance in *C. elegans*, but again, it was not the focus of this study. For this reason, we added a paragraph to the *Discussion* to address future directions and additional genes that could be tested, including reference to the IVR6 and IVR10 strains.

  In the *Discussion*, we have altered the following sentence to include P-glycoproteins and have cited the James *et al.* 2009 manuscript.

> "Genes involved in ivermectin metabolism, such as ATP-binding cassette (ABC) transporters, cytochrome P450 enzymes, GABA receptors, P-glycoproteins, and other signaling proteins, have been implicated in ivermectin resistance though much more research needs to be done to determine their role in the MoR of ivermectin [67,72-74]."
>
> Additionally, we added a sentence at the end of the mentioned *Discussion* paragraph to say: "Overall, additional genes outside of the GluCl family could be involved in the MoR of ivermectin. However, functional studies, such as those conducted in the present study, are essential to elucidate the role each gene plays in ivermectin resistance."

We hope these comments address the reviewer's concerns.

4) Reviewer #3: The manuscript provides novel and detailed insight into the fitness effects upon mutation of genes associated with anthelmintic resistance in the model nematode C. elegans. Defining fitness costs helps predict potential mechanisms of resistance against ivermectin and albendazole in natural settings. The authors have measured multiple aspects of fitness in mutants strains, mostly in large experiments of which the results deepen our understanding of previously identified genes involved in anthelmintic resistance. Finally, the authors present a detailed discussion of the (potential) consequences of their results for parasitic nematode species. The manuscript is well-written and adds substantial experimental evidence for understanding mechanisms of anthelmintic resistance.

Thank you for the comment and summary.

<u>Part II – Major Issues: Key Experiments Required for Acceptance</u>

5) Reviewer #1: (No Response)

6) Reviewer #2: Figure 4: The authors state: "we did not see a significant difference in median animal length between the double GluCl mutant avr-14 and avr-15 or the triple GluCl mutant, as reported previously [60]". This is quite surprising because according to Dent et al. the triple mutant is supposed to be at least 40-fold more resistant than the double avr-14 and avr-15 mutant. Moreover, Glendinning et al. PLOS One, 2011, showed that the sensitivity to Ivermectin of the triple mutant strain DA1316 can be restored by re-expressing avr-14 or other GluCl subunits, which confirm Dent et al. results. One main difference between the present work and the experiments conducted by Dent at al. is that here the authors performed their assays in liquid media and only with two doses of ivermectin. To draw definitive answers about the possible differential ivermectin sensitivity between the double and the triple mutants, could the authors perform Larval development assays with multiple ivermectin doses (dose/response) both on NGM plates and liquid media? This should confirm either their original results or the observations made by Dent et al.

Thank you for the comment. We have addressed the reviewer's comments here:

- The reviewer stated that the difference between the Dent *et al.* 1999 work and the work presented here was that Dent *et al.* performed survival assays on NGM plates at a range of concentrations and that we performed our HTA at two concentrations. Although the differences stated by the reviewer are true, there are ***many*** differences between the assays performed in the Dent *et al.* manuscripts and what was reported here that could account for the variation reported in our results. In the *Introduction*, we have revised the following paragraph to point out the major differences between the Dent *et al.* assays as stated here:

- "In *C. elegans*, GluCls are thought to be homopentameric or heteropentameric transmembrane complexes where six genes encode GluCl subunits: *avr-14*, *avr-15*, *glc-1*, *glc-2*, *glc-3*, and *glc-4* [63–66]. Although it is established that GluCl subunits are the main targets of ivermectin in *C. elegans*, null mutations in *avr-14*, *avr-15*, or *glc-1* individually do not cause ivermectin resistance [66]. However, mutagenesis studies of *C. elegans* showed that a triple GluCl mutant strain (*avr-14; avr-15 glc-1*) displayed greater than 4000-fold resistance and that a double GluCl mutant strain (*avr-14; avr-15*) had intermediate levels of resistance as compared to the wild-type strain [66]. This study demonstrated that mutations in multiple GluCl subunit genes can cause high-level ivermectin resistance. Nevertheless, it is essential to note that the mutagenesis studies in *C. elegans* were not performed in a controlled background and assessed only one trait, survival, as measured by placing embryos on agar plates with ivermectin and observing the number of embryos that grew to adulthood [64,66]. Because many genes, as shown by others [66–74] and outlined below, can cause ivermectin resistance, a controlled genetic background is essential. The Dent study performed screens in 10 ng/ml of ivermectin on Nematode Growth Medium (NGM) plates with animals in an *avr-15* mutant background.

  Comparing the Dent study with our current investigation, it is essential to note several key methodological differences. The Dent study performed EC_{37} estimates, which encompassed a range of ivermectin concentrations from 0.6 ng/ml (0.5 nM) to 13.8 ng/ml (12 nM) for strains without Dyf mutations. By contrast, our plate-based assays were conducted at 1.5 nM ivermectin, which does fall within the range of ivermectin concentrations used in the Dent assays. However, the Dent study allowed embryos (and subsequent generations of worms) to grow for two weeks, which would allow developmentally delayed animals to reach maturity as *C. elegans* completes its life cycle in four days. Moreover, assessing multiple traits to evaluate ivermectin resistance is important because GluCls are widely expressed across several tissues in the *C. elegans* nervous system and pharynx [67,75]. Survival is an aggregate trait that integrates developmental rate, fecundity, numerous behaviors, and metabolism. To adequately evaluate the MoR to ivermectin, fitness, development, and fecundity should be used to understand how GluCl subunit genes interact and play a role in resistance and, ultimately, assess how they could affect the spread of resistance alleles in parasite populations."

- Prior to selecting concentrations for liquid assays (and plate-based assays), we performed a dose response with each drug. For ivermectin, specifically in the liquid-based (HTA) assay, we performed a dose response at the following concentrations (0 nM, 125 nM, 250 nM, 500 nM, 1000 nM, 2500 nM, 5000 nM, 10000 nM) to select our final concentrations to use in the HTA. Concentrations of 250 nM and 500 nM were selected because they were the highest concentrations that we could use that showcased differentiation in growth between the most ivermectin-resistant strain (*avr-14*, *avr-15*, and *glc-1*) and the N2 control strain. Dosages of ivermectin higher than 500 nM were highly detrimental to all strains. Note that much higher concentrations of ivermectin are necessary for the liquid-based assays because animals are exposed to the drug for a total of 48 hours, whereas animals on the plate-based competition assays are exposed to drugs for a total of seven weeks or seven days in the fecundity assay. In the Dent *et al.* survival assays, animals were exposed to ivermectin on NGM plates for ~ two weeks. The length of time animals were exposed to the drug for each assay dictates the concentration needed to produce an observable effect.

- Additionally, we reviewed the concentration of ivermectin animals were exposed to in the Dent *et al.* 1999 manuscript. Dent *et al.* performed screens in 10 ng/ml of ivermectin on NGM plates with animals in an *avr-15* mutant background. The molecular weight of ivermectin is 875.1 g/mol, which means Dent *et al.* would have exposed their animals to an ivermectin concentration of ~ 8.751 nM. Additionally, the authors performed EC_{37} estimates, which encompassed a range of concentrations from 0.6 ng/ml (0.5 nM) to 13.8 ng/ml (12 nM) (for strains without Dyf mutations). Our plate-based assays (NGMA) were conducted at 1.5 nM ivermectin, which does fall within the range of ivermectin concentrations used in the Dent assays. Again, the length of time animals were exposed to ivermectin in our HTA is a much shorter time period than what was done in the Dent experiments, our competitive fitness assays, and our fecundity assays, which dictates the concentration of drug needed to elicit a response. Details comparing the Dent *et al.* study with the present study are integrated into the response above.

7) Reviewer #3: - Mutations (avr-14, avr-15 and glc-1) in the GluCl units of C. elegans were created in the strain PD1074, but were experimentally compared to the N2 strain and ben-1 mutant in the N2 background. Although the PD1074 is derived from the N2 strain, it is not completely clear how they (may) differ in fitness and whether this could affect the experiments by the authors. The authors should clarify based on either previous results / literature (like they explicitly do for strain PTM229) or their own experimental data if and how differences between N2 and PD1074 may affect interpretation of results.

Thank you for the thoughtful comment. Although few genetic differences between PD1074 and N2 likely exist, we do not have evidence that there are genetic background differences that would affect the results of this study. Importantly, PD1074 is a derivative of the Horvitz lab N2 strain separated by 3-5 generations, and the Andersen lab N2 strain is one generation removed from the Horvitz lab N2 strain. Yoshimura *et al.* 2019 describe that the PD1074 strain assembly had a 99.98% identity with the N2 strain, and that there are a total of 116 structural discrepancies between the two strains. Over 98% of the N2 strain's genes encoded unchanged products in the PD1074 strain, and a total of ~ 53 new genes are predicted in the PD1074 strain. In past published works, we have classified PD1074 by its isotype name in the *Caenorhabditis* Natural Diversity Resource (CaeNDR) database, N2. In CaeNDR, strains that are > 99.97% genetically identical are grouped into isotypes. PD1074 and N2 are nearly genetically identical. Therefore, in previous manuscripts, we chose to label PD1074 as N2. Here, we keep the separate designation of PD1074 and N2 so readers can know the genetic background in which the deletion strains were created should they want to request and use those strains.

We have also added more context to highlight the similarities between N2 and PD1074 in the *Methods* under "Generation of *ben-1* and GluCl deletion strains". This paragraph now reads:

- "Nine *Caenorhabditis elegans* deletion strains generated from an N2 background were used in this study (**Table S1**). The *ben-1* deletion mutant strain was generated in the N2 background using CRISPR-Cas9 genome editing [49,107]. The *avr-14*, *avr-15*, and *glc-1* single deletion mutant strains were generated in the PD1074 background using CRISPR-Cas9 genome editing by SunyBiotech (Fujian, China). The PD1074 strain is a lineal descendent of the laboratory-adapted N2 Bristol strain and is >99.97% genetically identical to the N2 strain, with 116 structural discrepancies between the two strains [108]. Over 98% of the N2 genes encoded unchanged products in PD1074 [108]. The N2 and PD1074 strains are grouped into the same isotype because of the high degree of genetic similarity [41,109]. The double and triple GluCl deletion mutant strains were generated by crossing the single GluCl deletion strains."

8) Line 706 (HTA method) describes that wells with fewer than 5 animals were excluded from the analysis. This is logical in terms of acquiring median and mean lengths which is not possible when too few or no animals are present. However, if embryos of highly sensitive strains do not hatch, biologically relevant information may be excluded from the assay. Principally this data could also be extracted by readers from Table S2. Although it's highly appreciated the raw data is included with the manuscript, it would help to summarize information about (potential) overrepresentations of excluded strains.

Thank you for the comment. The reviewer brings up two important points that we clarify here.

First, the reviewer asks about issues in drug sensitive strains. In the HTA, animals are placed into 96-well plates as embryos and allowed to hatch over 24 hours. After 24 hours, we check that all animals from each strain have hatched and are arrested at the L1 larvae stage prior to proceeding with the assay. If embryos have hatched and arrested at the L1 stage, we feed and drug the animals. Therefore, sensitive strains start at the same developmental stage as resistant strains, so it is not a difference in hatching rate as the reviewer states. We have edited the *Methods* to address this first point as follows:

- Lines 986-990: "After 24 hours, every plate was inspected to ensure that all embryos hatched and animals were developmentally arrested at the first larval (L1) stage to ensure all strains started each assay at the same developmental stage. Next, food was prepared to feed the developmentally arrested L1 animals using the required number of OD_{600}100 HB101 aliquots (see *Nematode food preparation for liquid culture assays*)."

Second, the reviewer asks about our exclusion of wells with fewer than five animals. As the reviewer pointed out, Table S2 contains data for the full HTA, which entails the mean worm length (µm), median worm length (µm), n (number of animals in a given well), and minimum worm length (µm), among other statistical values. We hope Table S2 provides transparency of the data that went into our analysis.

However, we do agree that further clarification is needed about our analysis metrics. We have edited the following sections of the *Methods* to address this concern:

After the description of metrics one through four in lines 1086-1090. We have added the following sentence:

"Importantly, all four steps described above were implemented to ensure all data were cleaned (*i.e.*, outliers removed), and we obtained balanced assays and complete strain and condition representation for all strains in the HTA. Using this process, we retained data from every assay, bleach, and condition for each strain, which highlights that all strains have appropriate representation in the analysis (**Table S2**)."

We hope this addition helps readers know that the data went through our model selection, cleaning steps, and that we removed measurements that were not representative of 80% of the data to ensure complete strain and condition representation. We placed the above explanation before our description of assay regression and normalization so reader's know those steps happen prior to statistical analysis.

We hope these points address the reviewer's concerns.

<u>Part III – Minor Issues: Editorial and Data Presentation Modifications</u>
<u>Reviewer #1:</u>

9) Line 89 'However, anthelmintic resistance has emerged' - clarify this is in veterinary helminths with only reduced efficacy reported in human helminths.

Thank you for the comment and the opportunity to provide clarification. We have rewritten this sentence to read:

"However, anthelmintic resistance has emerged in veterinary helminths, with reports of reduced drug efficacy against nematodes in humans, which threatens our ability to control parasitic nematode infections [4–8]."

10) 96 – 'when a single anthelmintic has reduced efficacy' – misleading as combination treatment needs to be initiated while both anthelmintics are highly effective (in veterinary helminths, combination treatments have not been effective after resistance has emerged)

Thank you for the comment. We have rewritten this sentence to read:

"Simultaneous treatment with two or more drugs (*i.e.*, co-administration) from different anthelmintic classes is one method used to slow the development of resistance [12-15]."

11) 99 – 'despite the successes of both strategies' – combination treatment and rotation are both widely used in livestock and horses but have failed to control resistance

Thank you for the comment. We have rewritten this sentence to read:

"Despite the implementation of both strategies, co-administration and anthelmintic rotation have failed to control resistance and pose the risk of multidrug resistance [9,19,20], particularly if a shared mechanism confers resistance to both drugs (*e.g.*, drug export) [21], a phenomenon known as cross-resistance [22]."

12) 104 – 'however, neither co-administration nor rotation alone are enough to slow the spread of resistance' – I don't believe we have the data to support this statement, we only know this approach fails when resistance to one or more of the anthelmintics is already present – co-administration may be effective approach to preserve new anthelmintics

Thank you for the comment. We have removed the sentence.

13) 168 – add 'at these particular loci'

Thank you for the suggestion. We have rewritten this sentence to read:

"Fourth, we sought to identify any evidence of cross-resistance between albendazole and ivermectin by comparing the fitness costs of each deletion mutation at *ben-1*, *avr-14*, *avr-15*, and *glc-1* in both drugs."

14) 222 – 'incredibly' to 'severely'

Thank you for the comment. We have rewritten this sentence to read:

"These results suggest that, when not under drug selection pressure, loss of *avr-15* is severely detrimental to animal fitness."

15) Figs 2 and 3 – helpful to add drug concentrations to legend

Thank you for the comment. Because Figures 2 and 3 have no legends , we have done the following to address this comment:

- Added the concentrations of each condition in parentheses after each condition is listed in the figure. We have changed the right axes labels to read:
    - Fig 2B: “DMSO (1%)”
    - Fig 2D: “Albendazole (1.25 µM)”
    - Fig 2F: “Ivermectin (1.5 nM)”
    - Fig 3A: “DMSO (1%)”
    - Fig 3B: “Albendazole (1.25 µM)”
    - Fig 3C: “Ivermectin (1.5 nM)”

Additionally, we have denoted the concentrations of each condition in Fig 2. and Fig 3. in the figure captions.

The Fig 2. caption now reads:

- “A barcoded N2 wild-type strain, PTM229, was competed with strains that have deletions in either one, two, or three genes that encode GluCl channels or the beta-tubulin gene *ben-1* in **(A)** DMSO (1%), **(C)** albendazole (1.25 µM), and **(E)** ivermectin (1.5 nM). Generation is shown on the x-axis, and the relative allele frequencies of the nine strains with genome-edited alleles and N2 are shown on the y-axis. The $\log_2$-transformed competitive fitness of each allele is plotted in **(B)** DMSO (1%), **(D)** albendazole (1.25 µM), and **(F)** ivermectin (1.5 nM)...”

The Fig 3. caption now reads:

- “Bar plots for lifetime fecundity, y-axis, for each deletion strain on the x-axis in **(A)** DMSO (1%), **(B)** albendazole (1.25 µM), and **(C)** ivermectin (1.5 nM) are shown...”

16) 285 – 325 fecundity data was very variable – is it possible the drug concentrations chosen were slightly too low to show a clear phenotype (e.g. no decrease in fecundity in N2 reference for either drug) and is there any possibility the regular transfers of hermaphrodites to new plates could reduce their viability/fecundity in a variable manner depending on transfer technique? The many conclusions from these experiments were quite unexpected and sometimes contradictory (e.g. avr-15 and avr15:avr14 LOF mutants were less fecund overall but both were significantly more fecund than controls and all other lines from days 5-7 in all conditions), which makes it difficult to assess if the interpretations are robust.

Thank you for the comment.

We did perform a dose-response on NGMA plates for each condition prior to selecting albendazole and ivermectin concentrations to use for this assay. In our plate-based dose-response assays, we picked 10 L4 individuals of each strain to a given NGMA plate and assessed the concentration that was needed for each condition to allow the animals to starve out the plate after one week. The reason that we decided on this metric is because the generation time for the competitive fitness assays is one week, and all strains needed to be exposed to a dose of drug that they could survive when exposed. It was necessary to pick a concentration that would allow animals the opportunity to survive so we could assess how alleles spread under these conditions. To keep both plate-based assays consistent, we exposed our fecundity assay animals to the same concentration of albendazole and ivermectin as done in the competitive fitness assay.

We have added two supplemental figures to show our dose-response results in ivermectin, because this drug has the most sensitive range compared to albendazole. First, we added a figure of strain PHX2502 (*avr-15*), the strain with the most delayed growth in ivermectin response. Second, we added a figure of the strain ECA2690 (*avr-14 avr-15*; *glc-1*) the fastest-growing strain in ivermectin. These two figures show why we selected a dosage of 1.5 nM ivermectin to use in the NGMA plate-based assays.

We have also added a section to the *Methods* under "Anthelmintic dose-response assays" to clarify how we selected our concentrations for each condition:

"Before selecting anthelmintic doses to use in the competitive fitness and fecundity assays, a dose response was performed to determine the appropriate dosage of albendazole and ivermectin for the given assay. For the dose response assays, gravid hermaphrodites were picked and placed into a bleach spot. After 24 hours, L1 larvae were picked to a new 6 cm NGMA plate. After 48 hours, ten late-stage L4 larvae hermaphrodites were picked to NGMA plates in the below conditions. Animals were exposed to albendazole at the following concentrations (µM): 0 (1% DMSO), 0.65, 1.25, and 2.5. Animals were exposed to ivermectin at the following concentrations (nM): 0 (1% DMSO), 1.2, 1.3, 1.4, 1.5, 1.6, 1.7, 1.8, 1.9, 2, 2.1, 2.25, 2.5, 5, and 10. Animals were imaged every day for seven days. After seven days in each condition, NGMA plates were checked for starvation. The highest concentration of each condition that allowed the most sensitive strain (**S9 Fig.**) to starve after seven days (*e.g.*, 1.5 nM ivermectin) compared to the most resistant strain (**S10 Fig.**) was selected to use in competitive fitness and fecundity assays. Using the described strategy to select the anthelmintic dosage allowed sensitive strains to compete against the wild-type control strain, PTM229, in the competitive fitness assays. The same anthelmintic dosage was used in the competitive fitness and fecundity assays to keep all NGMA-based assays consistent"

It is possible that the hermaphrodites get damaged upon transfer to a new NGMA plate. However, we ensured hermaphrodites were alive and had normal movement throughout each day of the experiment. If a hermaphrodite were to die or appear to be injured in the experiment, that replicate was not included in the results. We set up ten technical replicates of each strain in each condition to ensure that we had enough reproducibility for each strain and condition. In the *Methods*, we state, "Replicates where the original hermaphrodite parent died were excluded from the analysis of lifetime fecundity. Only biological replicates with data from all five assay plates were used to calculate daily and total fecundity (**Table S7**)." In terms of regular transfers reducing viability/fecundity, we have no evidence that transferring hermaphrodites affects the animal's offspring production. Although a more difficult to interpret dataset, we believe that we have enough replicates to adequately report the results from this fecundity assay and have followed protocols used in the field [68, 94, 95].

17) 338 - 340 unclear what this statement means

Thank you for the comment. The statement was meant to convey that the nine strains vary in length. However, all animals did grow to the expected size of an L4 animal. We have rewritten this sentence to read:

"Despite substantial variation among the strains in control (DMSO) conditions, animal measurements were categorized as the L4 larval stage by our custom CellProfiler worm models [82,83], indicating all strains developed to an appropriate length for animals in control conditions."

We hope this rewrite provides clarification.

18) 376 – 380 points are valid but could this be phrased better?

Thank you for the suggestion. We have rewritten this section and hope this rewrite provides more clarity. The section now reads:

"However, it is important to note that a loss of *ben-1* did confer a slight advantage compared to the N2 strain in the ivermectin fecundity assay (**Fig. 3C**). All GluCl deletion mutant strains, except the *glc-1* deletion mutant strain, conferred a slight advantage compared to the N2 strain in the albendazole HTA (**Fig. 4A**). However, it is essential to note that these slight advantages across conditions could be caused by genes targeted by both drugs expressed in the same tissues or cells. Importantly, the competitive fitness assays did not display any evidence of cross-resistance (**Fig. 2**)."

19) 418 – 419 'to identify why GluCls in parasites lack variation' what does this mean and please add references

Thank you for the comment. Citations have been added, and the sentence now reads:

"To identify why GluCls in parasites lack mutations in ivermectin-resistant isolates and why the field has yet to validate ivermectin-resistant genes [91], we tested the contributions of three genes that encode GluCl subunits on *C. elegans* fitness."

We have also added appropriate citations, as the reviewer suggested, that highlight the lack of identification of mutations in GluCls in ivermectin-resistant parasites:

Redman E, Sargison N, Whitelaw F, Jackson F, Morrison A, Bartley DJ, et al. Introgression of ivermectin resistance genes into a susceptible Haemonchus contortus strain by multiple backcrossing. PLoS Pathog. 2012;8: e1002534. doi:10.1371/journal.ppat.1002534

20) 430 'than what was tested here' rephrase

Thank you for the comment and suggestion. We have rewritten this sentence to now read:

"However, we focused on the effects of multi-generational competitive fitness, fecundity, and development when nematodes are exposed to ivermectin. By contrast, previous studies measured nematode response against different macrocyclic lactones (*i.e.*, abamectin), focused on different traits such as body bends, paralysis, and gene expression, and used different genetic approaches to assess resistance [92,93]."

We hope this rewrite provides clarity and addresses the reviewer's concern.

21) 447 – 448 could add 'but more recent genome-wide analyses have confirmed that mutations in GluCl subunit genes are not implicated in ivermectin resistance in Haemonchus'

Thank you for the comment. We agree with the reviewer's comment. We cited a paper by Doyle *et al.* that does not find that GluCls are associated with ivermectin resistant *H. contortus,* but it is important to note that the authors of this manuscript used a single isolate that was used for bulk-segregant analysis, and although GluCl mutations were not found in this isolate, it does not necessarily mean that GluCl mutations might not be associated with other *H. contortus* isolates. We have edited this section to read:

"A polymorphism in an *avr-14* ortholog has been identified in *Cooperia oncophora* [57]. Although polymorphisms in several genes (*Hco-glc-3* and *Hco-glc-5*) have been identified in resistant isolates of *Haemonchus contortus* [26,95-97], recent genome-wide analyses have not identified mutations in GluCl subunit associated in ivermectin resistant in *H. contortus* [98]."

22) 453 'lack of variation' again needs clarified and references

Thank you for the comment. We have edited the sentence and added appropriate citations. The sentence now reads:

"Second, the absence of mutations in GluCl genes in ivermectin-resistant parasites has led researchers to search for additional genes outside this gene family involved in the MoR of ivermectin [91]."

23) 467 paper cited does not show ivermectin resistance involves 'various genes and mechanisms'

Thank you for the comment and the opportunity to provide clarification. We have updated our citations to support the sentence. The sentence now reads:

"To date, ivermectin resistance across *Nematoda* appears to involve various genes and mechanisms [66,68-70]"

The updated citations referenced are:

Dent JA, Smith MM, Vassilatis DK, Avery L. The genetics of ivermectin resistance in Caenorhabditis elegans. Proc Natl Acad Sci U S A. 2000;97: 2674–2679. doi:10.1073/pnas.97.6.2674

Laing R, Doyle SR, McIntyre J, Maitland K, Morrison A, Bartley DJ, et al. Transcriptomic analyses implicate neuronal plasticity and chloride homeostasis in ivermectin resistance and response to treatment in a parasitic nematode. PLoS Pathog. 2022;18: e1010545. doi:10.1371/journal.ppat.1010545

Prichard RK. Ivermectin resistance and overview of the Consortium for Anthelmintic Resistance SNPs. Expert Opin Drug Discov. 2007;2: S41-52. doi:10.1517/17460441.2.S1.S41

Reyes-Guerrero DE, Jiménez-Jacinto V, Alonso-Morales RA, Alonso-Díaz MÁ, Maza-Lopez J, Camas-Pereyra R, et al. Assembly and analysis of Haemonchus contortus transcriptome as a tool for the knowledge of ivermectin resistance mechanisms. Pathogens. 2023;12: 499. doi:10.3390/pathogens12030499

We hope these edits address the reviewer's concerns.

24) 489 – 490 needs rephrased

Thank you for the comment and suggestion. We believe the reviewer is referring to the sentence that spans 730-731: "Previous research has shown that *ben-1* is highly expressed in cholinergic neurons, which causes BZ susceptibility [40].

We have edited the sentence to now read, " Previous research has shown that *ben-1* function in neurons, specifically in cholinergic neurons, underlies susceptibility to BZs [105]."

25) 496 'the overlap in expression in the same neurotransmitters' does not make sense

Thank you for the comment and suggestion. We have rephrased this sentence to read:

"The overlap of *ben-1* and GluCl gene expression in the same neuronal cell subtypes could explain the small advantages that *ben-1* conferred in ivermectin and the GluCls in albendazole in the HTA (**Fig. 4**)."

26) 514 – 515 'our understanding of ivermectin's MoR remains incomplete because we have not identified all the genes involved in ivermectin resistance' need to add 'in C. elegans, or any genes involved in ivermectin resistance in parasitic nematodes'

Thank you for the suggestion. We have made the following edit to the referenced statement:

"Nevertheless, our understanding of ivermectin's MoR remains incomplete because we have not identified all the genes involved in ivermectin resistance in *C. elegans* nor identified the genes involved in ivermectin resistance in parasitic nematodes."

Reviewer #2:

27) In the introduction, the paragraph on the co-administration of two or three drugs (lanes 95-101) seems to me to be oversold, and the bibliographical references mentioned do not fully support the authors' assertions.

Thank you for the suggestion. We have edited the following sections of the paragraph with specific changes in **bold**:

"Simultaneous treatment with two or more drugs (*i.e.*, co-administration) from different anthelmintic classes is one method **proposed** to slow the development of resistance [12–15]. Anthelmintic rotation, another **suggested** method to slow resistance, uses the periodic switching of drug classes to alleviate selection pressures on one drug class and prolong drug lifespan and efficacy [16–18]."

"Despite the **implementation** of both strategies, co-administration and anthelmintic rotation **have failed to control resistance** and pose the risk of multidrug resistance [9,19,20], particularly if a shared mechanism confers resistance to both drugs (*e.g.*, drug export) [21], a phenomenon known as cross-resistance [22]. We have also double-checked all citations in the referenced paragraph to ensure all citations back our claims.

We hope these edits address the author's concerns.

28) Lane 112: The claim that Bz and ML are "regularly Co-administrated" seems to be an overstatement. The authors give an old reference that do not support this claim".

Thank you for the comment. We have removed the word "regularly" and rephrased to address the reviewer's concern so the sentence now reads: "Two of the most commonly used anthelmintic drugs in MDA programs are albendazole, a benzimidazole (BZ), and ivermectin, a macrocyclic lactone (ML) [23-26], where research suggests that these two drugs can be co-administered as a chemotherapeutic treatment for intestinal helminths and lymphatic filariasis [27-30]."

Additionally, we have added more recent citations that better support this claim. The references cited are:

Beach MJ, Streit TG, Addiss DG, Prospere R, Roberts JM, Lammie PJ. Assessment of combined ivermectin and albendazole for treatment of intestinal helminth and Wuchereria bancrofti infections in Haitian schoolchildren. Am J Trop Med Hyg. 1999;60: 479–486. doi:10.4269/ajtmh.1999.60.479

Gandasegui J, Onwuchekwa C, Krolewiecki AJ, Doyle SR, Pullan RL, Enbiale W, et al. Ivermectin and albendazole coadministration: opportunities for strongyloidiasis control. Lancet Infect Dis. 2022. doi:10.1016/S1473-3099(22)00369-3

Palmeirim MS, Hürlimann E, Knopp S, Speich B, Belizario V Jr, Joseph SA, et al. Efficacy and safety of co-administered ivermectin plus albendazole for treating soil-transmitted helminths: A systematic review, meta-analysis and individual patient data analysis. PLoS Negl Trop Dis. 2018;12: e0006458. doi:10.1371/journal.pntd.0006458

Patel C, Hürlimann E, Keller L, Hattendorf J, Sayasone S, Ali SM, et al. Efficacy and safety of ivermectin and albendazole co-administration in school-aged children and adults infected with Trichuris trichiura: study protocol for a multi-country randomized controlled double-blind trial. BMC Infect Dis. 2019;19: 262. doi:10.1186/s12879-019-3882-x

29) Lane 368: The authors claim that: "Because albendazole and ivermectin are routinely distributed together to at-risk populations, it is critical to ensure that the two drugs do not have the same MoR to avoid the possibility of cross-resistance". To my knowledge, this practice is not very widespread. Can the authors provide appropriate bibliographical references to back up their assertions?

Thank you for the comment. We have edited the sentence to be more clear. The sentence now reads: "Because albendazole and ivermectin can be distributed together to at-risk populations to treat lymphatic filariasis and has been proposed as a strategy to treat soil-transmitted helminth infections [28–30,84,85], it is critical to ensure that the two drugs do not have the same MoR to avoid the possibility of cross-resistance." "

Additionally, we added the following publications to support this sentence:

Gandasegui J, Onwuchekwa C, Krolewiecki AJ, Doyle SR, Pullan RL, Enbiale W, et al. Ivermectin and albendazole coadministration: opportunities for strongyloidiasis control. Lancet Infect Dis. 2022. doi:10.1016/S1473-3099(22)00369-3

Palmeirim MS, Hürlimann E, Knopp S, Speich B, Belizario V Jr, Joseph SA, et al. Efficacy and safety of co-administered ivermectin plus albendazole for treating soil-transmitted helminths: A systematic review, meta-analysis and individual patient data analysis. PLoS Negl Trop Dis. 2018;12: e0006458. doi:10.1371/journal.pntd.0006458

Matamoros G, Sánchez A, Gabrie JA, Juárez M, Ceballos L, Escalada A, et al. Efficacy and safety of albendazole and high-dose ivermectin coadministration in school-aged children infected with Trichuris trichiura in Honduras: A randomized controlled trial. Clin Infect Dis. 2021;73: 1203–1210. doi:10.1093/cid/ciab365

McPherson S, Solomon AW, Seife F, Solomon H, Gebre T, Mabey DCW, et al. Pharmacokinetics, feasibility and safety of co-administering azithromycin, albendazole, and ivermectin during mass drug administration: A review. PLoS Negl Trop Dis. 2023;17: e0011224. doi:10.1371/journal.pntd.0011224

30) Figure 2E: Could the authors give an explanation to the sharp decrease in allele frequency after one day for both the avr-14 and avr-15 and the triple mutant strain in the competitive fitness assay carried in presence of ivermectin? Also, this is very surprising to observe fixation only two days later for these strains.

Thank you for the comment and the opportunity to clarify this point.

First, the x-axis for figures Fig 2A, Fig 2C, and Fig 2E displays generations, not days. Each generation on the x-axis is one week (*i.e.*, seven days). Therefore, each competition lasted for a total of seven generations (*i.e.*, seven weeks). Fixation for the *avr-14* and *avr-15* double mutant strain and the triple mutant strain were observed after two generations (*i.e.*, two weeks). To provide more clarity, we provide the following edits:

- We clarified that a generation is one week in the *Methods* under "Competitive fitness assays" and state: "Plates were grown for roughly one week to starvation (*i.e.*, one generation)."
- Importantly, the *avr-14* and *avr-15* double mutant strain and the triple mutant strain (along with all strains that have a loss of *avr-15*) drop in allele frequency in all three conditions (DMSO, albendazole, and ivermectin) as seen in Fig 2A, Fig 2C, and Fig 2D. To describe these observations, we have the following statements:
    - In regards to control conditions: "Notably, all strains with a loss of *avr-15*, whether it be a single, double, or triple GluCl mutant, were vastly unfit and ceased to exist in the population by the third generation (**Fig. 2A**). These results suggest that, when not under drug selection pressure, loss of *avr-15* is severely detrimental to animal fitness. Therefore, it is improbable that *C. elegans* strains with *avr-15* loss-of-function alleles will be observed in natural populations. Similarly, we do not expect *avr-15* loss-of-function alleles to be found in parasite populations.
    - In regards to albendazole exposure: "Notably, the loss of one, two, or all three GluCl genes caused a reduction of fitness in albendazole conditions, and each strain displayed a similar competitive fitness response in albendazole as observed in control conditions (**Fig. 2C, Fig. 2D**)."
    - In regards to ivermectin exposure: "The loss of *avr-14* or *avr-15*, the loss of both *avr-14* and *glc-1*, or the loss of both *avr-15* and *glc-1* caused significantly reduced competitive fitness in ivermectin compared to the control strain (**Fig. 2E, Fig. 2F**)."

31) Figure 3: I agree with the authors that: "Because strains with a loss of both avr-14 and avr-15 and a loss of all three GluCl subunits have significantly reduced fecundity across all conditions, it would be unlikely for animals in nature to acquire loss-of-function mutations in these genes that cause detrimental fitness consequences". However, could the authors explain why fecundity (Figure 2) and comparative fitness (Figure 3) are sometimes correlated and other times not? Are strain life-cycles affected by mutations or treatments?

Thank you for the comment and the opportunity to clarify. First, we answer the reviewer's question regarding if the strain life-cycles are affected by mutations or treatment. The only assay where animals are allowed to complete their life cycle is in the competitive fitness assay and life cycles do appear to be affected by both mutation and treatment. For example, we see in DMSO control conditions that animals with a loss of both *avr-14* and *avr-15* or a loss of all three GluCls are removed from the population by the third generation because of the effects of the mutations. We see that the N2 strain does perfectly fine in the competition assays in control DMSO conditions. On the other hand in ivermectin conditions, we see that animals with a loss of both *avr-14* and *avr-15* or a loss of all three GluCls sweep to fixation. Therefore, by being in the presence of a drug, animals with a loss of both *avr-14* and *avr-15* or a loss of all three GluCls are able to overcome the fitness consequences those mutations carry in control conditions and persist in the population when in the presence of a drug. We hope this answers the reviewer's question regarding life cycles affected by either mutations or treatments where a fitness balance between ivermectin's effects and the effects of GluCl loss.

Second, we have added a paragraph in the *Discussion* that highlights our hypothesis of why we observe differences between the three main assays: competitive fitness, fecundity, and development. We hope this new addition addresses the reviewer's question.

“Prior to this study, minimal research had been performed to assess the quantitative contributions of GluCl subunits on different traits involved in nematode fitness. To identify why GluCls in parasites lack mutations in ivermectin-resistant populations [91] and why we have yet to validate ivermectin-resistant genes [82], we tested the contributions of three genes that encode GluCl subunits on *C. elegans* fitness. We found that the competitive fitness and development assays produced similar results, but the fecundity assays deviated from the results in these two assays. We have two hypotheses to explain this discrepancy between assays. First, the competitive fitness assay focuses on two traits involved in nematode fitness: development (*i.e.*, time to reproduction) and reproductive rate. Because competitive fitness encompasses development, we can expect results from the competitive fitness assay and development assay to be more comparable than results of the fecundity assay. In the fecundity assay, we measured the daily and total fecundity of individual animals over seven days and did not focus on reproductive rate. Therefore, it is difficult to compare the daily fecundity of animals for the duration of one generation to the competitive fitness assay, where animals have offspring over multiple generations. Second, it is possible that mutations in *ben-1* or the GluCl subunit genes do not affect fecundity to the same extent that they affect development, rendering the fecundity results less clear to interpret compared to the competitive fitness and development assays. ”

32) Lane 521: The authors state: “our data suggest that cross-resistance is unlikely because ben-1 and the GluCl subunits do not appear to target the same MoR in C. elegans.” It's not discussed well enough. Indeed, it has been well documented in parasitic nematodes that multi-resistant isolates carry the individual mutations characteristic of each resistance type (for example see Doyle et al., Cell Reports, 2022).

Thank you for the comment. We have added a sentence to the *Discussion* that clarifies the Reviewer's concern.

“Finally, when considering the opportunity for multi-drug resistance to occur, either by cross-resistance or independent selection, our data suggest that cross-resistance is unlikely because *ben-1* and the GluCl subunits do not appear to target the same MoR in *C. elegans*. In studies of *H. contortus*, multi-drug resistant isolates carry independent mutations in genes specific to a given resistance phenotype, suggesting that albendazole and ivermectin target different genes [98]. Studies across *C. elegans* and *H. contortus* suggest that animals acquire multi-drug resistance independently and not by cross-resistance.”

Reviewer #3:

33) Control conditions in the experiments were performed under DMSO conditions as this is the solvent for the anthelmintic drugs. DMSO is reported to have clear effects on fitness of C. elegans (e.g. reproduction and growth). In case there is an interaction between a investigated mutation and DMSO, this may have consequences for the interpretation of the results. For example line 292 it is indicated loss of ben-1 suggestively leads to loss of fecundity in control conditions and in line 377 it is indicated ben-1 deletion leads to a advantage under ivermectin conditions, but this was also true under control DMSO conditions. Was the fitness of the mutants also (previously) tested under standard conditions without DMSO to enable extraction of DMSO effects?

We appreciate the Reviewer’s concern about the effect of DMSO on *C. elegans* in our assay. All of our assays that assessed drug response (*i.e.*, competitive fitness, fecundity, and development) exposed all animals to 1% DMSO for both control and drug (*i.e.*, albendazole and ivermectin) conditions.

The reviewer is correct that DMSO can have effects on *C. elegans* fitness. However, these effects have only been observed when animals are exposed to DMSO at higher concentrations (*e.g.*, above 2%). We have added the following sentence to our *Methods* section titled *Anthelmintic stock preparation for competitive fitness assays, fecundity assays, and HTAs*:

"All control conditions for every assay exposed animals to 1% DMSO, a concentration shown not to affect *C. elegans* normal physiology and lifespan [110,111]."

We also cited the following two manuscripts that looked at the effects of DMSO on *C. elegans* physiology.

110. AlOkda A, Van Raamsdonk JM. Effect of DMSO on lifespan and physiology in C. elegans : Implications for use of DMSO as a solvent for compound delivery. MicroPubl Biol. 2022;2022. doi:10.17912/micropub.biology.000634

111. Katiki LM, Ferreira JFS, Zajac AM, Masler C, Lindsay DS, Chagas ACS, et al. Caenorhabditis elegans as a model to screen plant extracts and compounds as natural anthelmintics for veterinary use. Vet Parasitol. 2011;182: 264–268. doi:10.1016/j.vetpar.2011.05.020

We hope this edit addresses the reviewer's concern.

34) Line 56 (and a few other similar cases in the manuscript): "… ivermectin resistance required loss of two GluCl genes (avr-14 and avr-15) or loss of three GluCl genes (avr-14, avr-15, and glc-1)." The sentence is not specific as resistance is already conferred by avr-14 and avr-15 it is not clear why glc-1 is also mentioned in terms of requirement.

Thank you for the comment. Because no statistical significant difference between the double deletion *avr-14* and *avr-15* and the triple mutant (*avr-14*, *avr-15*, and *glc-1*) in the competitive fitness or HTA in ivermectin conditions is observed, we have edited the manuscript to state that only a loss of *avr-14* and *avr-15* are required for ivermectin resistance.These changes were made in the *Introduction* and *Discussion*, the only two places where we talked about requirement.

35) Line 57: "The fecundity assays revealed that loss of ben-1 did not provide any fitness benefit in albendazole and that no GluCl deletion mutants were resistant to ivermectin." Should 'in albendazole' be 'in albendazole conditions'?

Thank you for the suggestion. We have revised the sentence to read: "The fecundity assays revealed that loss of *ben-1* did not provide any fitness benefit in albendazole conditions and that no GluCl deletion mutants were resistant to ivermectin."

36) Line 62: The text could clarify to which drug classed albendazole and ivermectin belong.

Thank you for the suggestion. We have identified which classes albendazole and ivermectin belong to at the start of the abstract. The first sentence of the abstract now reads:

"Albendazole (a benzimidazole) and ivermectin (a macrocyclic lactone) are the two most commonly co-administered anthelmintic drugs in mass-drug administration programs worldwide."

37) Line 138 and onwards: There are six GluCl subunits described, but it is not clarified why the manuscript focuses on three of the six mutations. In line 161 it then says all three GluCl subunits which can be interpreted as all six known GluCl subunits.

Thank you for the suggestion. We focused on the three mutations described because those genes are the three GluCls that have been most implicated in ivermectin resistance in the literature. We have changed our language throughout the manuscript to make it clear that we focused on three GluCl subunits and did not study all six GluCl subunits. Please see our changes throughout the manuscript.

Additionally, we have added a sentence to the *Introduction* that states:

"We focused on the three genes encoding GluCl subunits that have been most implicated in ivermectin resistance to date: *avr-14*, *avr-15*, and *glc-1*."

We also have a line in our *Discussion* that states:

"Nevertheless, our understanding of ivermectin's MoR remains incomplete because we have not identified all the genes involved in ivermectin resistance in *C. elegans* nor identified the genes involved in ivermectin resistance in parasitic nematodes. To solve this problem, we need to conduct additional experiments that quantitatively assess the fitness effects of all six GluCl subunit genes singly and in combination, both in control and ivermectin conditions."

We hope these changes address the reviewer's concerns.

38) Figure 3: A horizontal line at the height of the N2 reference would facilitate comparing N2 to the mutant strains.

Thank you for the comment and suggestion. We have incorporated a horizontal line at the height of the N2 reference strain to help facilitate the comparison between N2 and the mutant strains in Fig 3.

39) Figure 4: A and B y-axis titles differ, but based on the legend they should be the same.

Thank you for the comment and for finding this error. The reviewer is correct. The axis titles should be the same. We have now updated the y-axis to read "Normalized Animal Length (μm)" for both Fig 4A and Fig 4B. We also ensured the y-axis titles for S7 Fig also read "Normalized Animal Length (μm)," so all HTA figures are comparable.

40) Line 415: The authors argue that because GluCl mutations have significant fitness disadvantages when not exposed to ivermectin, these mutations are unlikely occur in nature. Nevertheless, ivermectin can remain detectable after drug administration for weeks to months and in some settings anthelmintic drug are frequently applied and manure from dewormed animals is spread over fields. Along the same line of reality in anthelmintic drug applications, it would be interesting to discuss the longer lifecycle of parasitic nematodes here. Longer life cycles of parasitic nematodes will result in more frequent exposure of generations to anthelmintic drugs.

Thank you for the comment and for bringing up this interesting point, but it is a difficult comment to address because we do not have long-term data about the effects of residues in hosts or in fields. However, we have tried to address the reviewer's critique and have added a

section to our *Discussion* to touch on the author's comments. The new section of the *Discussion* reads:

"On the contrary, animals under strong selection because of prolonged ivermectin exposure might select resistance alleles in GluCl genes, which would allow mutations in GluCl subunits to spread throughout populations. However, parasitic nematodes must remain under constant ivermectin exposure at higher than therapeutic doses to overcome the severe fitness consequences that mutations in GluCl subunits carry for selection to remain significant. Because human drug treatment programs apply ivermectin once to three times annually [88], it is unlikely that ivermectin persists in humans between treatments at concentrations that would cause constant drug selection pressure and subsequent selection of resistance alleles in parasitic nematodes. Although veterinary medicine prescribes ivermectin more frequently, ivermectin again is unlikely to remain at concentrations high enough to cause selective pressures, especially during winter months when treatment subsides [89,90]."

We hope this addition to the *Discussion* addresses the reviewer's comment.

41) Line 632: The subtitle could be a bit more informative to guide the reader and differentiate between the subtitle below.

Thank you for the suggestion. We have changed the subtitle from "High-throughput assays (HTAs)" to "High-throughput assays (HTAs) to assess nematode development." We hope this edit provides clarity.

42) Line 719: The linear model describes 'Bleach', which is not the most intuitive term for batch for people not working with C. elegans.

Thank you for the comment. Under *Methods* in the section "High-throughput Assays (HTAs), we have a few sentences that describe the role of bleach in our assays:

"Populations of each strain were amplified and bleach-synchronized for three independent assays. Independent bleach synchronizations controlled for variation in embryo survival and subsequent effects on developmental rates that could be attributed to bleach effects."

To help make the term 'bleach' clear in our linear model, we have edited the following sentence in the Methods:

"For each anthelmintic drug, we estimated a linear model using the raw phenotype measurement as the response variable and both assay and technical replicate identity as explanatory variables following the formula *median_wormlength_um ~ assay + bleach* (which accounts for bleach effects during bleach synchronization) using the *lm()* function in base R."

We hope this change adds clarity.

43) Line 727 and further: Different concentrations are used per anthelmintic drug. Were these standard concentrations or how did the authors decide on these concentrations?

Thank you for the comment. We have added a section in the *Methods* called "Anthelmintic dose-response assays" to describe how each anthelmintic dosage was selected. This section reads:

"Before selecting anthelmintic doses to use in the competitive fitness and fecundity assays, a dose response was performed to determine the appropriate dosage of albendazole and ivermectin for the given assay. For the dose-response assays, gravid hermaphrodites were

picked and placed into a bleach spot. After 24 hours, L1 larvae were picked to a new 6 cm NGMA plate. After 48 hours, ten late-stage L4 larvae hermaphrodites were picked to NGMA plates in the below conditions. Animals were exposed to albendazole at the following concentrations (μM): 0 (1% DMSO), 0.65, 1.25, and 2.5. Animals were exposed to ivermectin at the following concentrations (nM): 0 (1% DMSO), 1.2, 1.3, 1.4, 1.5, 1.6, 1.7, 1.8, 1.9, 2, 2.1, 2.25, 2.5, 5, and 10. Animals were imaged every day for seven days. After seven days in each condition, NGMA plates were checked for starvation. The highest concentration of each condition that allowed the most sensitive strain (**S9 Fig.**) to starve after seven days (*e.g.*, 1.5 nM ivermectin) compared to the most resistant strain (**S10 Fig.**) was selected to use in competitive fitness and fecundity assays. Using the described strategy to select the anthelmintic dosage allowed sensitive strains to compete against the wild-type control strain, PTM229, in the competitive fitness assays. The same anthelmintic dosage was used in the competitive fitness and fecundity assays to keep all NGMA-based assays consistent."

Additionally, we added two supplemental figures (S9 Fig and S10 Fig) to show nematode response in ivermectin in NGMA plates, the condition most questioned by reviewers. We hope this provides clarity.
